# Geometric target margin strategy of proton craniospinal irradiation for pediatric medulloblastoma

**DOI:** 10.1093/jrr/rrae066

**Published:** 2024-09-15

**Authors:** Takaaki Yoshimura, Keigo Kondo, Takayuki Hashimoto, Kentaro Nishioka, Takashi Mori, Takahiro Kanehira, Taeko Matsuura, Seishin Takao, Hiroshi Tamura, Takuya Matsumoto, Kenneth Sutherland, Hidefumi Aoyama

**Affiliations:** Department of Health Sciences and Technology, Faculty of Health Sciences, Hokkaido University, Sapporo 060-0812, Japan; Department of Medical Physics, Hokkaido University Hospital, Sapporo 060-8648, Japan; Global Center for Biomedical Science and Engineering, Faculty of Medicine, Hokkaido University, Sapporo 060-8648, Japan; Department of Health Sciences, School of Medicine, Hokkaido University, Sapporo 060-0812, Japan; Global Center for Biomedical Science and Engineering, Faculty of Medicine, Hokkaido University, Sapporo 060-8648, Japan; Global Center for Biomedical Science and Engineering, Faculty of Medicine, Hokkaido University, Sapporo 060-8648, Japan; Department of Radiation Oncology, Faculty of Medicine, Hokkaido University, Sapporo 060-8648, Japan; Department of Medical Physics, Hokkaido University Hospital, Sapporo 060-8648, Japan; Department of Medical Physics, Hokkaido University Hospital, Sapporo 060-8648, Japan; Faculty of Engineering, Hokkaido University, Sapporo 060-8638, Japan; Department of Medical Physics, Hokkaido University Hospital, Sapporo 060-8648, Japan; Faculty of Engineering, Hokkaido University, Sapporo 060-8638, Japan; Department of Radiation Technology, Hokkaido University Hospital, Sapporo 060-8648, Japan; Department of Medical Physics, Hokkaido University Hospital, Sapporo 060-8648, Japan; Global Center for Biomedical Science and Engineering, Faculty of Medicine, Hokkaido University, Sapporo 060-8648, Japan; Department of Radiation Oncology, Faculty of Medicine, Hokkaido University, Sapporo 060-8648, Japan

**Keywords:** proton beam therapy, medulloblastoma, treatment planning, craniospinal irradiation

## Abstract

In proton craniospinal irradiation (CSI) for skeletally immature pediatric patients, a treatment plan should be developed to ensure that the dose is uniformly delivered to all vertebrae, considering the effects on bone growth balance. The technical (t) clinical target volume (CTV) is conventionally set by manually expanding the CTV from the entire intracranial space and thecal sac, based on the physician’s experience. However, there are differences in contouring methods among physicians. Therefore, we aimed to propose a new geometric target margin strategy. Nine pediatric patients with medulloblastoma who underwent proton CSI were enrolled. We measured the following water equivalent lengths for each vertebra in each patient: body surface to the dorsal spinal canal, vertebral limbus, ventral spinal canal and spinous processes. A simulated tCTV (stCTV) was created by assigning geometric margins to the spinal canal using the measurement results such that the vertebral limb and dose distribution coincided with a margin assigned to account for the uncertainty of the proton beam range. The stCTV with a growth factor (correlation between body surface area and age) and tCTV were compared and evaluated. The median values of each index for cervical, thoracic and lumber spine were: the Hausdorff distance, 9.14, 9.84 and 9.77 mm; mean distance-to-agreement, 3.26, 2.65 and 2.64 mm; Dice coefficient, 0.84, 0.81 and 0.82 and Jaccard coefficient, 0.50, 0.60 and 0.62, respectively. The geometric target margin setting method used in this study was useful for creating an stCTV to ensure consistent and uniform planning.

## INTRODUCTION

Medulloblastoma is one of the most common malignant cancers of the central nervous system in pediatric patients. It occurs in the posterior fossa and has the potential for leptomeningeal metastasis [1, 2]. Medulloblastoma is diagnosed based on magnetic resonance imaging findings and confirmed by surgical histology and molecular features, as defined in the World Health Organization classification of tumors of the central nervous system published in 2021 [3, 4]. Tumors are categorized as low-, standard- or high-risk, based on the volume of postoperative residual tumors and the presence or absence of metastases.

Treatment outcomes for medulloblastoma have greatly improved by combining surgery, chemotherapy and radiotherapy [5]. Because medulloblastomas have the propensity to disseminate through the brain and spinal cord, craniospinal irradiation (CSI) plays an essential role in providing long-term disease control, with a clinical cure rate of approximately 70–5% [6–9]. Numerous comparisons of the dose distributions between conventional photon and proton beam therapy (PBT) for CSI have been reported. Compared with conventional X-ray therapy, proton CSI has clinical and physical advantages, such as sparing normal tissue from the radiation dose [10]. Multiple dosimetric studies have demonstrated that CSI with PBT delivers equivalent target dose volume coverage with significant dose reduction for organs at risk (OARs) anterior to the vertebral body compared with conventional X-ray therapy [11–13].

In particular, intensity-modulated proton CSI (ipCSI) using spot-scanning PBT is considered safer than passive scattering PBT for children and adolescents and young adults (AYAs) because it decreases the contamination of neutrons and improves the dose distribution at the junction of multiple fields [14–16]. Timmermann *et al.* reported that performing scanning-based proton CSI in pediatric patients with recurrent medulloblastoma minimized the ventral dose [17]. Stoker *et al.* performed intensity-modulated proton therapy (IMPT) simulations on 10 patients with medulloblastoma who had undergone conventional passively scattered proton therapy (PSPT) and reported improved robustness of the irradiation field junctions and greater preservation of OARs [15]. Giantsoudi *et al.* compared PSPT and IMPT treatment plans for two pediatric patients with medulloblastoma and reported reduced doses to the skin and esophagus and more intensive irradiation [16]. We also investigated the clinical outcomes of AYA patients who were treated with vertebral body-sparing ipCSI at our institution. We observed a lower incidence of serious acute hematological toxicity compared to CSI cases previously treated with conventional X-ray therapy [18]. Thus, PBT is expected to provide equivalent tumor control while reducing the adverse events caused by irradiation, thereby ensuring an improved and longer life for cancer survivors. However, because there is a concern that an excessive dose concentration may cause deformities due to growth disturbances, the irradiation dose should be carefully prescribed [19].

In the treatment planning of ipCSI for pediatric medulloblastoma, the clinical target volume (CTV) is generally contoured to include the entire intracranial space and thecal sac and total vertebral body, based on treatment planning guidelines [19]. In the current spinal target volume selection strategy, vertebral body-sparing ipCSI is used in AYA patients with skeletal maturity. On the other hand, it is necessary to avoid the risk of spinal deformity related to asymmetric irradiation across the vertebral body in growing children (under 15 years of age) [16, 20]. In a previous study, the asymmetric irradiation of the vertebral body resulted in scoliosis. Furthermore, the magnitude of this effect was related to the irradiation dose [21]. Thus, most radiation oncologists prescribe a uniform dose to the vertebral body and prefer to uniformly inhibit growth to avoid the risk of lordosis from asymmetric irradiation to the vertebral body. This is consistent with the Children’s Oncology Group guidelines in current clinical trials, including CSI, and is the recommendation of a recent European intergroup consensus statement [22].

However, to the best of our knowledge, there are no published guidelines concerning the extent to which the CTV should be extended to cover the vertebral body; the degree of expansion of the technical CTV (tCTV) depends solely on the experience of the physician, and even for the same physician, the criteria for creating tCTV change with experience. Medek *et al.* surveyed 28 radiation oncologists in the USA using 11 questions on practice patterns. They found that the vertebral body coverage in CSI with PBT varies among radiation oncologists with respect to target delineation, CTV expansion and OARs modifications [23]. All physicians who responded to the questionnaire indicated that they uniformly irradiated all vertebrae in pediatric patients with medulloblastoma; to irradiate all vertebrae uniformly in nonskeletally mature patients, the spinal canal was targeted, and the tCTV was expanded in the ventral-dorsal direction.

Considering the effects of bone growth balance, the isodose line of the prescribed dose should coincide as closely as possible with the vertebral body. However, robust coverage of the entire vertebral body within the CTV results in a higher dose distribution to the OARs located on the ventral side of the vertebral body, such as the lungs, heart and esophagus. Therefore, physicians selected a balanced treatment plan between the implemented vertebral body dose coverage and OARs dose sparing [23].

In this study, we hypothesized that if we could geometrically identify the extent to which the tCTV could be expanded from the spinal canal in the ventral-dorsal direction and mechanically create the tCTV, it would be possible to reduce the inter- and intra-operator differences caused by manual creation of the tCTV, increasing consistency among planners. We proposed a simple method for tCTV expansion using the vertebral body index measured on planned computed tomography (CT) images. This study aimed to demonstrate that the effectiveness of this method is independent of the physician’s experience.

## MATERIALS AND METHODS

### Patient data

This retrospective analysis included nine pediatric patients with medulloblastoma who had previously received ipCSI at our institution between December 2016 and August 2019. Patient characteristics are listed in Table 1. The median age at enrollment was 9 years (range: 5–11 years). This study was approved by the Ethics Committee of our hospital (019–0397). Written informed consent, including general consent, was obtained from the patient or a person with parental authority before treatment.

**Table 1:** Characteristics of the patients in this study

		*n*	(%)	Median	Range
Number of patients		9			
Age				9	5–11
Sex	Male	6	66.7%		
	Female	3	33.3%		
Height [cm]				130.4	115–148.5
Weight [kg]				21.9	18.5–32.45
BMI				14.1	12.5–18.2
BSA [${\mathrm{m}}^2$]		9	100%	0.90	0.80–1.13
Risk	Standard risk	5	55.6%		
	High	4	44.4%		

BMI, body mass index, BSA, body surface area

Generally, the following phase exists between the time of treatment-planning CT image acquisition and the start of treatment: contouring the target, normal tissue and tCTV by physician; dose calculation using the treatment planning system (TPS) by the medical physicist and implementation of patient-specific quality assurance by dosimetrists. In most cases, 10 business days are set aside for the start of treatment after the treatment-planning CT image acquisition. Within the limited machine operational time, approximately two or three days should be set aside for patient-specific quality assurance. We identified how much time each process took. We defined the time taken for contouring (${D}_c$) as the number of business days from the date of treatment-planning CT image acquisition until the date the data were transferred to the medical physicist, the time taken for treatment planning (${D}_p$) as the number of business days from the start of the treatment planning until the plan approval, the time taken for patient-specific quality assurance (${D}_q$) as the number of business days from the approval of the treatment plan until the completion of the patient-specific quality assurance, and the time taken for treatment preparation (${D}_t$) as the number of business days from the date of treatment-planning CT image acquisition until the treatment start.

### Treatment planning process

All patients underwent a treatment planning CT scan with a slice thickness of 2.5 mm using an Optima CT580W (General Electronic Healthcare, Waukesha, WI, USA). We used a synchrotron-based spot-scanning proton-beam system, PROBEAT-RT (Hitachi, Ltd., Tokyo, Japan), at our facility to deliver the treatments. The synchrotron beam had a maximum range of 30 g/cm^2^ and an irradiation field size of 30 × 40 cm. The details of our synchrotron-based spot-scanning proton-beam delivery system with a gating function have been provided in previous reports [24, 25]. The details of our ipCSI planning have also been reported previously [18]. In short, two laterally opposed fields are used for whole-brain irradiation, and one or two posterior fields are used for whole-spine irradiation using IMPT, with a relative biological effectiveness of 1.1. For patients under 15 years of age, the total vertebral body, whole intracranial space and entire thecal sac are contoured as the CTV [18]. The dose distribution in the CTV is expected to be as homogeneous as possible.

**Fig. 1:**
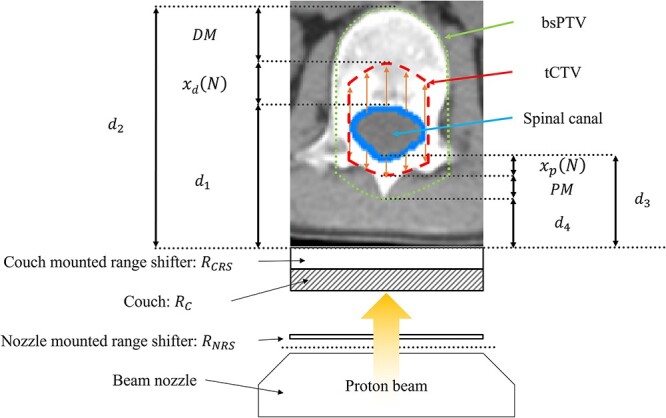
Overview of the geometric target margin strategy in this study. Thin orange arrows show the expansion from spinal canal to tCTV. tCTV: technical clinical target volume, bsPTV: beam-specific planning target volume, DM: distal margin, PM: proximal margin.

The required water-equivalent length (WEL) values were measured to identify the geometric margins using a VQA TPS (Hitachi, Ltd.). We defined each measurement value as follows (Table 2 and Fig. 1): ${d}_1$, distance from the skin to the distal spinal canal; ${d}_2$, distance from the skin to the distal vertebral body; ${d}_3$, distance from the skin to the proximal spinal canal and ${d}_4$, distance from the skin to the proximal spinous process. These were measured from the upper, middle and lower slices of each vertebra and the average value was calculated. In addition to the couch (${R}_c$: 7.8 mm WEL), some patients had a couch-mounted range shifter (${R}_{CRS}$: 32 mm WEL) and an additional nozzle-mounted range shifter (${R}_{NRS}$: 5 mm WEL) inserted at the time of treatment-planning CT imaging, which were considered when determining the geometric margin.

**Table 2:** Definition of the symbols and terms used in this study

Symbol	Definition
${D}_c$	The time taken for contouring as the number of business days from the date of treatment planning CT image acquisition until the data transferred to the medical physicist
${D}_p$	The time taken for treatment planning as the number of business days from the start of the treatment planning until the plan approval
${D}_q$	The time taken for patient-specific quality assurance as the number of business days from the approval of the treatment plan until the completion of the patient-specific quality assurance
${D}_t$	The time taken for treatment preparation as the number of business days from the date of treatment planning CT image acquisition until the treatment start
$BSA$	Body surface area
$CTV$	Clinical target volume
$tCTV$	Technical CTV in clinical practice, which is contoured by one physician
$stCTV$	Simulated tCTV in this study
$PTV$	Planning target volume
$bsPTV$	Beam specific PTV
$DM$	Distal margin
$PM$	Proximal margin
${d}_1$	WEL from surface to distal end of the spinal canal
${d}_2$	WEL from surface to distal end of the vertebral column
${d}_3$	WEL from surface to proximal end of the spinal canal
${d}_4$	WEL from surface to proximal end of the vertebral column
${R}_c$	WEL of couch (7.8 mm WEL)
${R}_{NRS}$	WEL of nozzle-mounted range shifter (5 mm WEL)
${R}_{CRS}$	WEL of couch-mounted range shifter (32 mm WEL)
$N$	The index number of vertebral body
$g$	Growth factor
${x}_d(N)$	WEL of technical distal margin from distal end of the Nth spinal canal
${X}_d(N)$	${x}_d(N)$ converted to the real thickness
${x}_p(N)$	WEL of technical proximal margin from proximal end of the Nth spinal canal
${X}_p(N)$	${x}_p(N)$ converted to real thickness
${X}_d\left(N,g\right)$	${X}_d(N)$ with growth factor
${X}_p\left(N,g\right)$	${X}_p(N)$ with growth factor
$dH\left(A,B\right)$	Hausdorff distance is the average over all slices of the largest value of the smallest distance to agreement on each slice between tCTV and stCTV
$MDA$	Mean distance-to-agreement
$DSC\left(A,B\right)$	Dice similarity coefficient between tCTV and stCTV; a $DSC\left(A,B\right)$ of 1 implies a perfect agreement overlap; a $DSC\left(A,B\right)$ of 0 equals no agreement
$J\left(A,B\right)$	Jaccard similarity coefficient measured similarity between tCTV and stCTV

WEL, water equivalent length

Generally, the planning target volume (PTV) is created by adding geometric margins to the CTV to address the uncertainties in treatment delivery [26]. In a previous report, simple geometric expansions of the CTV were inadequate for PBT treatment planning, considering sensitivity to treatment uncertainties [27–29]. The beam-specific PTV (bsPTV) is therefore used to account for various uncertainties associated with the actual dose delivery, patient setup, organ motion and proton beam range calculation [30]. The bsPTV for pediatric patients with medulloblastoma was set to overlap with the vertebral body. The lateral margin of the bsPTV for CTV is 7.0 mm for spinal irradiation [18]. The distal margin and proximal margin are the beam-specific margins for the expansion of the CTV as an optimization volume of the bsPTV. The values were calculated as follows:


(1) \begin{equation*} DM={L}_d\times 3.5\%+1\ \mathrm{mm}\ \mathrm{WEL} \end{equation*}

(2) \begin{equation*} PM={L}_p\times 3.5\%+1\ \mathrm{mm}\ \mathrm{WEL}, \end{equation*}

where ${L}_d$ and ${L}_p$ represent the depths of the distal and proximal edges in the water-equivalent space, respectively. One millimeter was added to compensate for beam uncertainty in the depth direction [31, 32]. ${L}_d$ and ${L}_p$ were calculated as follows:


(3) \begin{equation*} {L}_d={d}_1+{x}_d(N)+{R}_c+{R}_{NRS}+{R}_{CRS} \end{equation*}

(4) \begin{equation*} {L}_p={d}_3-{x}_p(N)+{R}_c+{R}_{NRS}+{R}_{CRS}. \end{equation*}

The values obtained from the above equations were expressed in units of WEL. However, the WEL must be converted to the real thickness because the structures in MIM version 7.1.2 (MIM Software, Inc., Cleveland, OH, USA) were enlarged to the actual thicknesses. Here, ${x}_d(N)$ converted to the real thickness is denoted as ${X}_d(N)$, and ${x}_p(N)$ converted to the real thickness is denoted as ${X}_p(N)$. The relative stopping power was used to convert the WEL into the real thickness. For the relative stopping power, a conversion table representing the relationship between the CT values and relative stopping power registered in the treatment planning device, VQA TPS, was used. Conversion parameters used were 1.34 for the cervical spine (C-spine) and 1.27 for the thoracic (T-spine), lumbar (L-spine) and sacral vertebrae [33, 34].

Body size differs according to age. Based on the clinical growth chart for boys and girls published by the Japanese Society for Pediatric Endocrinology, height and weight increase with age [35, 36]. However, it is difficult to use age as a function of body size because of individual differences in growth. Therefore, we focused on body surface area (BSA) during chemotherapy, which was calculated as follows:


(5) \begin{equation*} BSA={W}^{0.425}\times{H}^{0.725}\times 0.007184 \end{equation*}

where $W$ is the patient weight and $H$ is the patient height. This formula, proposed by Du Bois *et al.*, has been used to calculate BSA in studies by the Japanese Clinical Oncology Group (JCOG), since the JCOG0501 trial [37]. We examined the correlation between BSA and age. We took this correlation as a correction function ($f(g)$) for growth at ${X}_d(N)$ and ${X}_p(N)$ as follows:


(6) \begin{equation*} {X}_d\left(N,g\right)={X}_d(N)\times f(g) \end{equation*}

(7) \begin{equation*} {X}_p\left(N,g\right)={X}_p(N)\times f(g) \end{equation*}

We expanded the structure of the spinal canal for simulated tCTV (stCTV) by adding our correction function for growth as the growth factor.

### Evaluation

Correlation coefficients and coefficients of determination were used to determine if there was a correlation between the expanded WEL for each vertebra [38, 39]. Correlation coefficients (*r*) were used to estimate geometric margins of the vertebrae. Previous studies have shown that 0 ~ ±0.1 indicates no correlation, ±0.1 ~ ±0.39 indicates a weak correlation, ±0.4 ~ ±0.69 indicates a correlation, ±0.7 ~ ±0.89 indicates a strong correlation, and ± 0.9 ~ ±1.0 indicates a very strong correlation [39]. The coefficient of determination (*R*^2^) was calculated for each vertebra. Harsdorf distance [$dH\left(A,B\right)$], mean distance-to-agreement (MDA), Dice similarity coefficient [$DSC\left(A,B\right)$] and Jaccard similarity coefficient $[J(A,B)$] were used to compare the simulated stCTV created in this study with the tCTV used in clinical practice [40–43].

In this study, the tCTV was contoured by one physician and is used in clinical practice as the gold standard. The stCTV in this study was modeled to approximate the tCTV in clinical practice. If both stCTV and tCTV completely matched, $DSC\left(A,B\right)$ and $J\left(A,B\right)$ of 1.0, were assigned. If the $DSC\left(A,B\right)$ between the stCTV and tCTV was >0.75, the proposed method for the geometric margin of the tCTV was defined as successful, following the convention often used in deep learning-based object detection and semantic segmentation. We compared the stCTV and tCTV for each patient with or without the growth factor taken into account by using $dH\left(A,B\right)$, $MDA$, $DSC\left(A,B\right)$ and $J\left(A,B\right)$ for the whole spine, C-spine, T-spine, L-spine and S-spine, respectively. Moreover, we evaluated the tCTV and stCTV volumes with or without the growth factor. The Wilcoxon signed-rank test was used for statistical comparisons between the tCTV and both stCTVs. Statistical analyses were performed using JMP Pro 16 software (SAS Institute Inc., Cary, NC, USA). Statistical significance was set at *P* < 0.05.

We predicted the trends of geometric margins with respect to each vertebral body. The area of the predicted distance [${X}_d(N)$ and ${X}_p(N)$] plus the spinal canal was defined as the stCTV without the growth factor; similarly, [${X}_d\left(N,g\right)$ and ${X}_p\left(N,g\right)$] plus the spinal canal were defined as the stCTV with the growth factor; and the area of the stCTV plus the path length $\times$3.5% + 1 mm was outlined as the bsPTV using the MIM software.

## RESULTS

The mean and range values of ${D}_c$, ${D}_p$ and ${D}_q$ were 2.7 [2–4], 6.3 [4–9] and 2.6 [2, 3] business days, respectively. The mean and range value of ${D}_t$ was 11.6 [10–13] business days. It should be noted that these data do not purely reflect the time spent on each process, which were performed concurrently with other clinical tasks in their respective occupations.

**Fig. 2:**
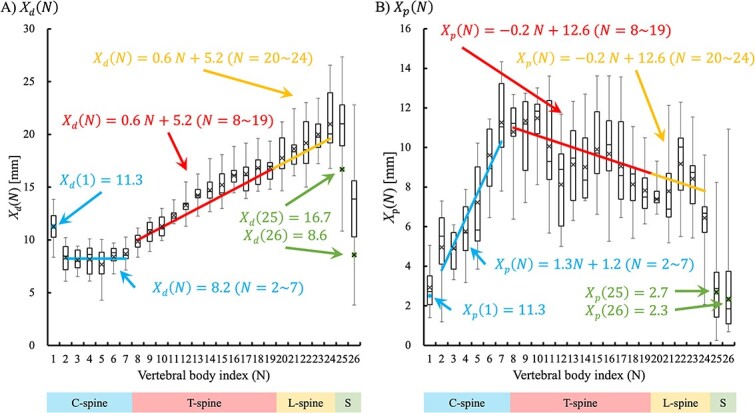
The relationship of $\boldsymbol{{X}_d(N)}$ and $\boldsymbol{{X}_p(N)}$ for each vertebral body index (*N*).

Each vertebral body distance was measured and the calculated expanded WEL $[{x}_d(N)$ and ${x}_p(N)$] was compared for each vertebra, as shown in Table 3. The converted real thicknesses of ${x}_d(N)$ and ${x}_p(N)$ [${X}_d(N)$ and ${X}_p(N)$] for each vertebra are shown in Fig. 2. For ${X}_d(N)$, the cervical vertebrae excluding the atlas (C1) had a correlation coefficient of 0.34. Therefore, the cervical vertebrae, excluding the atlas, were estimated to be uncorrelated and the magnification distance was almost constant. The atlas was excluded because of the presence of the skull in the beam path, which may have affected the proton beam range more than other cervical vertebrae.

**Table 3:** Relationship between the calculated expanded WEL [$\boldsymbol{{x}_d(N)}$ and $\boldsymbol{{x}_p(N)}$] and *N*

		${x}_d(N)$ [mmWEL]					${x}_p(N)$ [mmWEL]				
	*N*	Mean	95% CI (−)	-	95% CI (+)	*R* ^2^	Mean	95% CI (−)	-	95% CI (+)	*R* ^2^
C-spine	1	15.1	13.2	-	17.1	0.08	3.9	2.7	-	5.1	0.13
	2	11.2	9.6	-	12.8	0.63	6.7	4.7	-	8.6	0.29
	3	10.9	9.8	-	12.0	0.60	6.6	5.3	-	7.9	0.39
	4	10.9	9.4	-	12.5	0.59	7.8	6.0	-	9.5	0.00
	5	10.3	8.5	-	12.1	0.65	9.7	6.4	-	13.0	0.07
	6	11.3	10.2	-	12.4	0.61	12.9	10.6	-	15.2	0.13
	7	11.7	10.7	-	12.6	0.57	15.1	13.0	-	17.3	0.08
T-spine	8	12.6	11.7	-	13.4	0.01	13.7	11.7	-	15.8	0.66
	9	13.7	12.6	-	14.9	0.29	14.5	11.3	-	17.6	0.37
	10	14.4	13.3	-	15.5	0.09	14.6	12.5	-	16.8	0.36
	11	15.7	15.0	-	16.4	0.00	12.8	10.0	-	15.6	0.61
	12	16.9	15.8	-	18.1	0.29	10.4	8.4	-	12.3	0.26
	13	18.1	16.9	-	19.3	0.07	11.6	9.6	-	13.7	0.25
	14	18.8	17.3	-	20.2	0.15	11.5	9.8	-	13.2	0.23
	15	19.4	17.9	-	20.9	0.34	12.6	10.1	-	15.1	0.35
	16	20.5	19.2	-	21.8	0.18	12.3	9.7	-	14.9	0.66
	17	20.7	18.9	-	22.6	0.31	11.5	9.6	-	13.4	0.58
	18	21.4	19.8	-	23.1	0.42	10.4	8.7	-	12.0	0.46
	19	21.5	19.9	-	23.1	0.43	10.0	8.8	-	11.2	0.28
L-spine	20	22.6	20.5	-	24.8	0.47	9.4	9.0	-	9.9	0.02
	21	23.8	21.6	-	25.9	0.35	9.9	8.0	-	11.8	0.36
	22	24.5	21.8	-	27.1	0.62	11.7	9.5	-	13.9	0.09
	23	25.5	23.3	-	27.6	0.26	10.7	8.9	-	12.6	0.01
	24	26.7	23.6	-	29.9	0.00	8.2	5.4	-	11.0	0.46
S-spine	25	26.2	21.3	-	31.1	0.61	3.4	1.8	-	5.1	0.24
	26	16.8	10.9	-	22.6	0.46	3.0	1.4	-	4.5	0.15

C-spine, cervical spine; CI, confidence interval; L-spine, lumbar spine; S-spine; sacral spine; T-spine, thoracic spine; WEL, water equivalent length; $x$_d_(N), WEL of technical distal margin from distal end of the Nth spinal canal; $x$_p_(N), WEL of technical proximal margin from proximal end of the Nth spinal canal

**Fig. 3:**
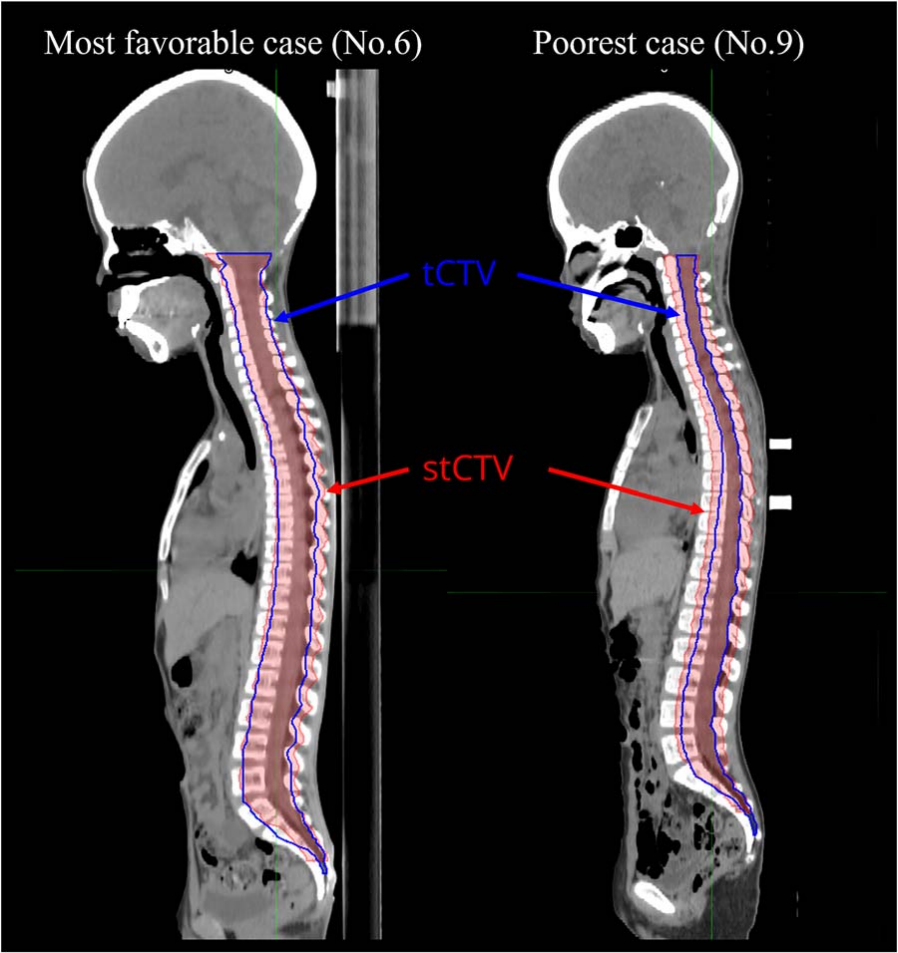
Example of the difference between tCTV and stCTV in the most favorable and poorest case. Although both tCTVs are contoured by the same physician, the degree of extension from the spinal canal varies with the physician’s experience. In the C-spine, the tCTV in case 6 is contoured over approximately half of the vertebral body, whereas the tCTV in case 9 is contoured over only in the spinal canal. Similarly, in the lumber spine, the tCTV in case 9 has a narrower extension range than that in case 6. tCTV: technical clinical target volume.

The thoracic and lumbar vertebrae had a strong correlation coefficient of 0.99. Because a proportional relationship was estimated for ${X}_d(N)$, an approximate linear line was established for the vertebral number $N$ (Fig. 2). In addition, the correlation coefficients for the thoracic and L-spine in ${X}_p(N)$ were − 0.77 and 0.19, respectively. Although the correlation coefficients for the T-spine in ${X}_p(N)$ had a strong negative correlation $\left(r=-0.77\right)$, the value of ${X}_p(N)$ in each T-spine showed a large variation and a small tilt compared with ${X}_d(N)$ in the T-spine. Thus, the magnified distance of the T-spine in ${X}_p(N)$ was estimated to be approximately constant. Therefore, it was approximated as a function of $N$ for the T-spine and L-spine in ${X}_d(N)$, C-spine in ${X}_p(N)$ and T-spine and L-spine in ${X}_p(N)$ as follows:


(8) \begin{equation*} {X}_d(N)=0.6\ N+5.2\ \left[\mathrm{mm}\right]\ \left(N=8\sim 24\right) \end{equation*}

(9) \begin{equation*} {X}_p(N)=\left\{\!\!\!\!\begin{array}{c}1.3\ N+1.2\ \left[\mathrm{mm}\right]\ \left(N=2\sim 7\right)\\ {}-0.2\ N+12.6\ \left[\mathrm{mm}\right]\ \left(N=8\sim 24\right).\end{array}\right. \end{equation*}

For ${X}_d(N)$, it was inferred that the C-spine had a constant value (C2 ~ 7: 8.2 mm), except for the atlas (C1), and that the thoracic and lumbar vertebrae had a proportional relationship.

For correction of growth, the median and range of BSA was 0.90 (0.80–1.13) m^2^. The correlation coefficient was 0.69, indicating a positive correlation. To calculate ${X}_d\left(N,g\right)$ and ${X}_p\left(N,g\right)$, $f(g)$ was expressed by the following equation:


(10) \begin{equation*} f(g)=0.052\times g+0.51, \end{equation*}

where $g$ is the patient age.

**Fig. 4:**
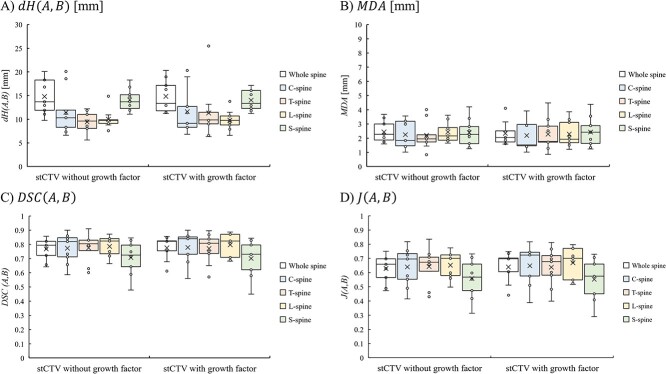
The variance of evaluation index for $ \boldsymbol{dH\left(A,B\right)}$, $\boldsymbol{MDA}$, $\boldsymbol{DSC\left(A,B\right)}$ and $\boldsymbol{J\left(A,B\right)}$ between tCTV and sTCV with or without growth factor.

The results of the comparison of the stCTV and tCTV with the geometric margin in the patients are shown in Fig. 3. Although both tCTVs were contoured by the same physician, the degree of extension from the spinal canal varied with the physician’s experience. As shown in Table 4, we compared the stCTV and tCTV for each patient with or without the growth factor taken into account by using $dH\left(A,B\right)$, $MDA$, $DSC\left(A,B\right)$ and $J\left(A,B\right)$ for the whole spine, C-spine, T-spine, L-spine and S-spine, respectively. When the growth factor was not considered, the median values and ranges of $dH\left(A,B\right)$, $MDA$, $DSC\left(A,B\right)$ and $J\left(A,B\right)$ for the whole spine were 13.70 (9.77–20.09) mm, 2.27 (1.59–3.68) mm, 0.79 (0.64–0.86) and 0.66 (0.47–0.75), respectively. The median values of $dH\left(A,B\right)$, $MDA$, $DSC\left(A,B\right)$ and $J\left(A,B\right)$ for the C-spine, T-spine, L-spine and S-spine were as follows: $dH\left(A,B\right)$, 10.32, 9.62, 9.77 and 13.70 mm; $MDA$, 1.84, 1.95, 2.16 and 2.26 mm; $DSC\left(A,B\right)$, 0.82, 0.80, 0.82 and 0.73 and $J\left(A,B\right)$, 0.70, 0.67, 0.70, 0.57, respectively. In addition, when the growth factor was considered, the median values and range of $dH\left(A,B\right)$, $MDA$, $DSC\left(A,B\right)$ and $J\left(A,B\right)$ for whole spine were 13.33 (11.21–20.31) mm, 3.20 (2.73–3.68) mm, 0.82 (0.61–0.86) and 0.54 (0.47–0.62), respectively. The median values of $dH\left(A,B\right)$, $MDA$, $DSC\left(A,B\right)$ and $J\left(A,B\right)$ for the C-spine, T-spine, L-spine and S-spine were as follows: $dH\left(A,B\right)$, 9.14, 9.84, 9.77 and 13.33 mm; $MDA$, 3.26, 2.65, 2.64 and 3.60 mm; $DSC\left(A,B\right)$, 0.84, 0.81, 0.82 and 0.73 and $J\left(A,B\right)$, 0.50, 0.60, 0.62 and 0.35, respectively. The variance of these data was shown in Fig. 4.

**Table 4:** Results of evaluation between stCTV and tCTV. A: without growth factor, B: with growth factor

A) Without growth factor								
	$dH\left(A,B\right)$ [mm]		$MDA$ [mm]		$DSC\left(A,B\right)$		$J\left(A,B\right)$	
	Median	Range	Median	Range	Median	Range	Median	Range
Whole spine	13.70	9.77–20.09	2.27	1.59–3.68	0.79	0.64–0.86	0.66	0.47–0.75
C-spine (C1–C7)	10.32	6.61–20.09	1.84	1.01–3.56	0.82	0.59–0.90	0.70	0.42–0.82
C-spine (C2–C7)	8.30	5.60–11.93	2.01	0.84–3.58	0.79	0.57–0.91	0.66	0.40–0.84
T-spine	9.62	5.60–12.21	1.95	0.84–4.01	0.80	0.60–0.91	0.67	0.43–0.84
L-spine	9.77	7.57–14.87	2.16	1.66–3.62	0.82	0.66–0.87	0.70	0.50–0.77
S-spine	13.70	11.07–18.30	2.26	1.26–4.21	0.73	0.48–0.85	0.57	0.31–0.73
B) With growth factor								
	$dH\left(A,B\right)$ [mm]		$MDA$ [mm]		$DSC\left(A,B\right)$		$J\left(A,B\right)$	
	Median	Range	Median	Range	Median	Range	Median	Range
Whole spine	13.33	11.21–20.31	3.06	2.44–3.68	0.82	0.61–0.86	0.55	0.47–0.62
C-spine (C1–C7)	9.14	12.70–20.31	3.26	2.96–3.56	0.84	0.56–0.90	0.50	0.42–0.58
C-spine (C2–C7)	8.30	5.60–12.32	2.66	1.75–3.58	0.83	0.54–0.91	0.54	0.40–0.84
T-spine	9.84	6.35–25.46	2.65	1.28–4.01	0.81	0.57–0.90	0.60	0.43–0.84
L-spine	9.77	6.59–13.76	2.64	1.93–3.36	0.82	0.68–0.89	0.62	0.56–0.77
S-spine	13.33	11.21–17.13	5.70	2.81–4.38	0.73	0.45–0.84	0.35	0.29–0.73

C-spine, cervical spine; $dH\left(A,B\right)$, Hausdorff distance; $DSC\left(A,B\right)$, Dice coefficient; $J\left(A,B\right)$, Jaccard coefficient; L-spine, lumbar spine; $MDA$, mean distance-to-agreement; S-spine, sacral spine; stCTV, simulated technical clinical target volume; T-spine, thoracic spine; tCTV, clinically used technical clinical target volume

**Fig. 5:**
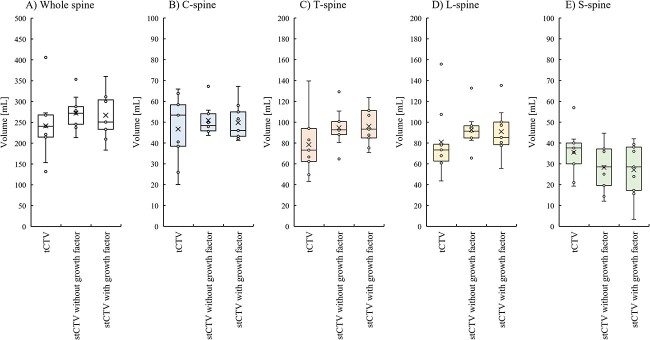
The variance of volume between tCTV and sTCV with or without growth factor.

As shown in Table 5, we also compared the tCTV and stCTV in this study. The median volume of the tCTV, stCTV without growth factors and stCTV with growth factors for the whole spine, C-spine, T-spine, L-spine and S-spine were as follows: whole spine, 240.68 ml, 272.03 mL (*P* = 0.19) and 250.84 ml (*P* = 0.43); C-spine, 53.37 ml, 48.37 ml (*P* = 0.72) and 46.03 ml (*P* = 0.72); T-spine, 73.1 ml, 92.68 ml (*P* = 0.19) and 93.31 ml (*P* = 0.11); L-spine, 73.41 ml, 91.43 ml (*P* = 0.09) and 85.32 (*P* = 0.19) and S-spine, 37.61 ml, 28.59 ml (*P* = 0.19) and 28.59 ml (*P* = 0.25), respectively. There was no significant difference between the volume of the tCTV and the stCTV in each category. The variance of these data was shown in Fig. 5.

## DISCUSSION

In recent years, proton CSI has been increasingly used because of dose distribution-sparing of OARs and improved availability of proton treatment facilities. The optimal extent of vertebral body coverage in proton CSI for skeletally immature patients is important because of concerns regarding asymmetrical growth and the risk of lordosis. In this study, calculated WEL values were required for the stCTV using treatment-planning CT images. The stCTVs were created using geometric margins and compared with the tCTVs by calculating $dH\left(A,B\right)$, $MDA$, $DSC\left(A,B\right)$ and $J\left(A,B\right)$. In this study, the median values of the $DSC\left(A,B\right)$ for the C-spine, T-spine, L-spine and S-spine considered with the growth factor were 0.82, 0.80 and 0.82, respectively. We also evaluated the tCTV and stCTV volumes. There was no significant difference between the volume of the tCTV and the stCTV in each category. Our results indicated a high degree of similarity between the tCTV and stCTV in each category. We demonstrated a method for creating a tCTV with individual spinal canal margins based on the vertebral body index. Although there was still a large variation in each indicator, the stCTV created using this method showed a high agreement with the physician’s manually contoured tCTV.

In this study, the geometric margin of the sacral vertebrae was calculated as the average geometric expansion distance for each patient. Unlike the other vertebrae, only two sacral vertebrae were measured, which meant that only 18 data points could be measured for the nine patients combined. Owing to the lack of data, no geometric margin was established for the sacral vertebrae in this study. Future studies should increase the number of data points to clarify the existence of this relationship.

As shown in Fig. 3, the tCTV was delineated differently in the most favorable and poorest cases. This indicates that there is variability in the creation of the tCTV among intraoperators. The $dH\left(A,B\right)$ and $MDA$ values were higher in the sacral spine than in the C-spine, T-spine and L-spine (Table 4). This may be due to differences in the way the tCTV was delineated. Therefore, by increasing the amount of data on the sacral vertebrae and inferring the relationship, it is possible to create a stCTV with smaller values of $dH\left(A,B\right)$ and $MDA$.

**Table 5:** Results of the volume of tCTV and stCTV in this study

Volume [ml]	Clinical ROI		Without growth factor		With growth factor	
	Median	Range	Median	Range	Median	Range
Whole spine	240.68	131.93–406.00	272.03	216.62–353.29	250.84	185.88–360.05
C-spine (C1–C7)	53.37	20.12–65.94	48.37	43.62–67.18	46.03	41.27–67.24
C-spine (C2–C7)	34.56	15.17–46.94	38.01	35.00–47.54	38.03	31.72–48.21
T-spine	73.13	43.11–139.63	92.68	64.71–129.23	93.31	70.76–123.74
L-spine	73.41	43.55–155.75	91.43	65.57–132.71	85.32	55.67–135.30
S-spine	37.61	19.32–56.99	28.59	14.39–44.74	28.59	12.85–42.06

C-spine, cervical spine; CTV, clinical target volume; L-spine, lumbar spine; S-spine; sacral spine; stCTV, simulated technical clinical target volume; T-spine, thoracic spine; tCTV, clinically used technical clinical target volume

In most cases, 10 business days are set aside for after the planning CT image acquisition and before the start of treatment. In this study, the mean time was 11.6 days. In addition, the number of trials in the treatment planning process is important to obtain better dose distribution in ipCSI. However, the dose calculation time per trial needs to be sufficiently long and only two or three trials can be performed per day owing to the vast irradiation area. Thus, to ensure a sufficient number of trials to obtain the desired dose distribution, there is a clinical need to reduce the number of trials by creating the tCTV and the contouring time. Since the tCTV was contoured manually, it took an average of 2.7 business days for ${D}_c$ in this study. However, our proposed method involves mechanically creating the tCTV. The time for creating the stCTV took less than an hour. Hernandez *et al.* validated an automated contouring tool using deep learning models for pediatric CSI [44]. In their results, 97% of the generated structures were scored as clinically acceptable, with 92% requiring no edits. By combining these new techniques, ${D}_c$ can be made shorter. This is expected not only to contribute to the reduction of ${D}_c$ but also to allow for an extension of the available time for ${D}_p$ within the limited time before the start of treatment.

In a previous study, Medek *et al.* assessed current practice patterns among radiation oncologists at US proton centers who treat pediatric patients to help understand how the optimal extent of vertebral body coverage influences outcomes when treating skeletally immature pediatric patients with proton CSI [23]. Based on a retrospective analysis, which included 146 patients receiving external beam radiation therapy between 1970 and 1977, Dörr *et al.* found a significant correlation between the incidence of kyphoscoliosis and the vertebral body dose gradient in patients under the age of 6 years [45]. Most radiation oncologists cover the entire vertebral body when treating skeletally mature patients. In addition, opinions on the anterior expansion of the CTV were divided among those who answered 3–4 mm or no expansion in the survey study by Medek *et al.* [23] These results were dependent on facility and physician policies. On the other hand, the most common choice in the aforementioned study was the plan where the vertebral body lay within the 80% isodose line in for patients aged 7 years or younger who underwent proton CSI [23]. Thus, there are no clear consensus guidelines for proton CSI covering the vertebral body in young pediatric patients. It is therefore hoped that creating a geometrical stCTV and proposing a certain vertebral body coverage will lead to standardization in proton CSI planning and reduce the workload of physicians.

This study had several limitations. The first was the limited number of patients from a single facility. The number of patients with medulloblastoma treated using CSI was limited because pediatric tumors are rare compared to other major cancers, such as prostate or lung cancers, and the number of pediatric patients in our facility is small [24]. Furthermore, verification using external data was insufficient. This requires the accumulation of a sufficient number of cases in a multicenter study.

Second, there was a great deal of variation within the creations by a single physician. In addition, based on the results of previous report by Madek *et al.*, it is clear that there is a great deal of variation even when the creation is conducted by multiple physicians [23]. It is necessary to discuss a large number of cases in a multicenter study to reduce this variability.

Third, the present study did not adequately consider differences in body size by age. Because the body sizes of the 3- and 15-year-old patients were very different, it is likely that correction for age is necessary when setting the geometric stCTV. Similarly, the height and weight of the patient may affect the setting of the geometric stCTV; therefore, corrections should be considered in future studies. Based on the growth standards for Japanese children with percentile values based on the national survey data of 2000, height and weight are increased with age [35]. Although our target patients did not deviate significantly from the growth standards, it is also true that bone size and muscle or fat content varies with age. Generally, there is a difference in physique based on sex and age. However, there were virtually no differences in growth in this study’s target age group between boys and girls, since this study did not include the AYA generation. Thus, we did not consider the sex difference in growth and rather focused on the correlation between BSA and age as growth factor in this study. As shown in Table 4, the use of this growth factor slightly improved the degree of region of interest agreement compared to that in cases where the growth factor was not used. Therefore, it is reasonable to use BSA as a growth factor. Nevertheless, there were large individual differences, and a large-scale study, such as the national survey data of 2000, may be needed to generalize the results of this study. Therefore, the number of cases must be increased to account for the influences of other factors. However, because pediatric medulloblastoma is classified as a rare cancer, it is difficult to collect data from the number of cases at a single institution. Therefore, collaboration with other institutions is necessary for further research. For example, Liu *et al.* performed a multi-institutional comparative analysis of proton and photon therapy-induced hematologic toxicity in patients with medulloblastoma. They enrolled 97 pediatric patients (60 who received PBT and 37 who received photon therapy) who underwent CSI without concurrent chemotherapy or concurrent single-agent vincristine [46].

The final limitation of this study was inter- or intra-operator variability, not only for the contouring of the tCTV by physicians but also for the measurement of ${d}_1$~${d}_4$. In this study, we visually and manually confirmed ${d}_1$~${d}_4$ in the upper, middle and lower slice planes of each vertebra using a single operator. Therefore, the acquired ${d}_1$~${d}_4$ values may have differed between inter- and intra-operators. As a result, the created stCTV may also have had inter- and intraoperator errors. However, this aspect of the proposed method has not yet been fully evaluated. Minimizing this error, for example, through automatic measurements in a large cohort, may be a future challenge. Moreover, even for the same observer, the method of creating a tCTV changed with experience. Therefore, the definition of the gold standard is very important, and even more important for artificial intelligence-based technologies for automatic contouring in radiation therapy compared to conventional approaches, such as atlas-based or image value thresholding [47].

In conclusion, the stCTV setting method proposed in this study generated volumes with a higher degree of agreement than the tCTV method used in clinical practice, demonstrating the usefulness of geometric margins. The stCTV setting method proposed in this study not only solved the problem of stCTV agreement accuracy among physicians, which was the aim of this study, but also significantly reduced the time required for stCTV creation. Therefore, our method demonstrated significant clinical benefits. However, further development of a technique to improve the accuracy of geometric stCTV agreement in pediatric patients with medulloblastoma is necessary.
